# Validation of an osteoporosis self-assessment tool to identify primary osteoporosis and new osteoporotic vertebral fractures in postmenopausal Chinese women in Beijing

**DOI:** 10.1186/1471-2474-14-271

**Published:** 2013-09-22

**Authors:** Yong Yang, BingQiang Wang, Qi Fei, Qian Meng, Dong Li, Hai Tang, JinJun Li, Nan Su

**Affiliations:** 1 Clinical Osteoporosis Center, Department of Orthopaedics, Beijing Friendship Hospital, Capital Medical University, Beijing 100050, China

**Keywords:** Beijing, Women, Osteoporosis, Osteoporosis screening tool for Asians, Validation, Fracture

## Abstract

**Background:**

This study aimed to validate the effectiveness of the Osteoporosis Self-assessment Tool for Asians (OSTA) in identifying postmenopausal women at increased risk of primary osteoporosis and painful new osteoporotic vertebral fractures in a large selected Han Chinese population in Beijing.

**Methods:**

We assessed the performance of the OSTA in 1201 women. Subjects with an OSTA index > -1 were classified as the low risk group, and those with an index ≤ -1 were classified as the increased risk group. Osteoporosis is defined by a T-score ≤ 2.5 standard deviations according to the WHO criteria. All painful, new vertebral fractures were identified by X-ray and MRI scans with correlating clinical signs and symptoms. We determined the sensitivity, specificity, and area under the receiver operating characteristic (ROC) curve for correctly selecting women with osteoporosis and painful new vertebral fractures.

**Results:**

Of the study subjects, 29.3% had osteoporosis, and the prevalence of osteoporosis increased progressively with age. The areas under the ROC curves of the OSTA index (cutoff = -1) to identify osteoporosis in the femoral neck, total hip, and lumbar spine were 0.824, 0.824, and 0.776, respectively. The sensitivity and specificity of the OSTA index (cutoff = -1) to identify osteoporosis in healthy women were 66% and 76%, respectively. With regard to painful new vertebral fractures, the area under the ROC curve relating the OSTA index (cutoff = -1) to new vertebral fractures was 0.812.

**Conclusions:**

The OSTA may be a simple and effective tool for identifying the risk of osteoporosis and new painful osteoporotic vertebral fractures in Han Chinese women.

## Background

With the progressive aging of populations, osteoporosis has rapidly become a growing global health concern because of its age-associated, exponentially increasing prevalence, morbidity, mortality, and costs [1]. Osteoporosis, which ultimately leads to fragility fractures, represents a major public health problem in Asian countries, especially China, which is the largest and most populous developing country in the world. It is projected that 50% of the world’s hip fractures, which contribute to the greatest amount of morbidity and mortality, will occur in Asian countries by the end of 2050 [2]. Once a person has developed osteoporosis or sustained fractures, restoration of full bone strength is unlikely because of irreversible structural loss in bone microarchitecture [3-5]. It is therefore very important to identify elderly populations at risk of developing osteoporosis and prevent the occurrence of the first fracture.

There is an important need in China for osteoporosis detection methodology. Measurement of bone mineral density (BMD) using dual X-ray absorptiometry (DXA) is widely accepted as the gold standard of osteoporosis diagnosis, and it is a primary predictor of fragility fractures. Unfortunately, DXA is not widely available in most developing countries including China because the DXA and BMD measurement equipment are expensive. There are currently several machines in some large third-tier general hospitals in Beijing, but there are no DXA machines in many cities in China. Hence, an ideal simple screening tool to identify the elderly population at risk for osteoporosis would not require DXA measurements.

Advanced age and low body weight are strongly associated with low BMD and with increased fracture risk [6-9]. The Osteoporosis Self-Assessment Tools for Asians (OSTA), which is based on age and body weight, has been found to be a good and simple tool with high sensitivity and acceptable specificity for the identification of women at risk of osteoporosis in previous studies [9-13]. However, another study reported poor results when validating use of the OSTA for identifying postmenopausal osteoporosis with lumbar spine DXA BMD measurements in a Chinese cohort [14]. The use of OSTA should be validated across diverse populations.

Because symptomatic osteoporotic vertebral fractures are associated with significant morbidity, mortality, and health and social service expenditures, it is important that vertebral fractures are detected early, and that the most appropriate treatments are administered as soon as possible. Percutaneous kyphoplasty (PKP) is a widely accepted, effective means of treating recently acquired painful osteoporotic vertebral fractures (new bone marrow edema on sagittal T1-weighted and fat-suppressed T2-weighted images in magnetic resonance imaging [MRI]), which can relieve pain quickly, increase stability immediately, and recover vertebral body height. Osteoporosis, with or without painless fractures, can treated effectively by pharmacological interventions. However, relatively few patients with painful vertebral fractures seek medical attention. Firstly, these patients often have no trauma history and they may not know that their acute back pain is associated with osteoporotic fracture. Moreover, many physicians without experience in a primary hospital or community healthy service center may also miss the diagnosis. Secondly, DXA and MRI are not widely available in most developing countries, including China. X-ray images do not reveal developmental variations in vertebral height (vertebral wedging and compression) and cannot be used to judge fracture age. As a result, patients with recently acquired painful vertebral fractures may not be diagnosed correctly and may not receive timely treatment. A good screening tool may be helpful for patients as well as for physicians with limited professional experience as an aide for identifying when additional attention is needed and for selecting the most appropriate therapy.

It remains to be confirmed whether OSTA could be used to identify recently acquired painful osteoporotic vertebral fractures. Therefore, the objective of this study was to validate the effectiveness of OSTA as an assessment tool for identifying postmenopausal women at increased risk of primary osteoporosis and recent painful osteoporotic vertebral fractures in a large selected Han Chinese population in Beijing.

## Methods

### Study population

The study population included postmenopausal Chinese women recruited from the osteoporosis clinic at the Beijing Friendship Hospital from September 2010 to October 2011. The study population included healthy women who came to the hospital for health examinations, and clinically symptomatic patients with a painful vertebral fracture within the past 6 months who came for further treatment. The inclusion and exclusion criteria are listed in Table 1. Subjects with abnormal biochemistry, including tests for renal and liver function, serum calcium, phosphate, total alkaline phosphatase, and thyroid-stimulating hormone, were also excluded. The study was approved by the Ethics Committee of Beijing Friendship Hospital, Capital Medical University and all subjects had signed the informed consent.

**Table 1:** Inclusion and exclusion criteria for this study

**Inclusion criteria**	**Exclusion criteria**
Postmenopausal for ≥12 months	A history or evidence of metabolic bone disease (e.g. type I diabetes, hyper- or hypoparathyroidism, Paget’s disease, osteomalacia, renal osteodystrophy, osteogenesis imperfecta)
Han Chinese nationality	Evidence of rheumatoid arthritis
Residency in Beijing for ≥20 years	History of organ transplantation
Willingness to participate in the study	The presence of cancer(s) with known metastasis to bone
Ability to read informed consent form	A long history of smoking or alcohol consumption
Ability to provide informed consent	Evidence of significant renal impairment
	Menopause before the age of 40 years
	Previous fracture or replacement of both hips
	The presence of a prolonged immobility condition (e.g., spinal cord injury, Parkinson’s disease, stroke, muscular dystrophy, ankylosing spondylitis)
	Prior use of an antiresorptive (e.g. bisphosphonate, estrogen, selective estrogen receptor modulators and calcitonin) or anabolic agent (e.g. fluoride and parathormone-PTH)

### BMD measurements and identification of recent clinical osteoporotic vertebral fractures

All women were invited to the osteoporosis clinic at Beijing Friendship Hospital for DXA BMD measurements of the hip and spine. The subjects were asked to fill in a questionnaire by a trained interviewer to provide demographic information and clinical risk factors for osteoporosis by referring to a structured table [15], including previous fragility fracture history, parental history of hip fracture, glucocorticoid treatment history (>5 mg prednisolone daily for 3 months or more), smoking, alcohol consumption, rheumatoid arthritis history, other secondary causes of osteoporosis mentioned in the exclusion criteria and medical treatment history. Height was measured with a stadiometer. Weight was measured without shoes in light indoor clothing using an electronic balance scale (accuracy, 0.1 kg).

The BMD, expressed in g/cm^2^, was measured with the use of the Hologic Discovery QDR Wi dencitometer (Hologic, Inc., MA, USA) on the lumbar spine (L1–L4) and left femur (femoral neck, trochanter, Ward’s triangle, and total hip). The *in vivo* short-term reproducibility values for the machine for postmenopausal women at the lumbar spine, femoral neck and total hip were all lower than 1%. The mean values from young Chinese women were used to calculate the T-scores: L1–L4 BMD 0.967 ± 0.11 g/cm^2^, femoral neck 0.803 ± 0.10 g/cm^2^, and total hip BMD 0.864 ± 0.11 g/cm^2^. All DXA measurements were performed by an experienced technologist.

Excluding healthy women who came for routine screening, all clinically symptomatic patients accepted further radiological examinations and treatments. We defined four requisite clinical criteria to confidently identify new painful osteoporotic vertebral fractures. These criteria were as follows: (1) postmenopausal status without trauma history or with a low-energy trauma history (low-energy trauma fracture was defined as a fracture resulting from a fall from a standing position or lower); (2) pain occurring within 6 months prior to BMD measurement; (3) acute or sub-acute vertebral fractures with correlating clinical signs and signs demonstrated by X-ray (i.e., height loss in the anterior, middle, or posterior dimension of a vertebral body that exceeds 20% of the vertebral body’s area in a lateral-view image of the thoracic/lumbar spine; or the presence of endplate deformities, a lack of parallelism of the endplates and a generally altered appearance relative to neighboring vertebrae) and spine MRI imaging (new bone marrow edema apparent in sagittal T1-weighted and fat-suppressed T2-weighted images); and (4) no history or evidence of metabolic bone disease or cancer. Patients with an atypical MRI manifestation were subjected to additional bone scanning. In this study, fractures were confirmed according to the above clinical criteria.

### OSTA

The OSTA [10] was calculated based on age and body weight using the following formula: [body weight (kg) - age (year)] × 0.2.

The decimal digits were then disregarded. As described in the original report [10], subjects with OSTA values ≤ -1 were classified as having an increased risk of osteoporosis and those with index values > -1 were considered to have a low risk for osteoporosis. For example, a 77-year-old woman whose body weight was 45 kg would have an OSTA index as calculated below: (45–77) × 0.2 = -6.4. The decimal digit (0.4) was then disregarded, and the OSTA index was equal to the integer -6. Therefore, this subject was classified as having an increased risk of osteoporosis.

### Statistical analysis

Osteoporosis is defined arbitrarily when any T-score (lumbar spine, femoral neck, or total hip) is -2.5 standard deviations (SD) or less, according to the World Health Organization (WHO) criteria. In this study, the characteristics of the OSTA risk index were examined at a T-score cutoff of -2.5 (osteoporosis), and the T-scores at individual BMD sites were used as outcome variables. The ability of OSTA to discriminate low BMD as defined by a T-score ≤ -2.5 was evaluated using receiver-operating characteristic (ROC) curve analysis, which plots sensitivity against (1–specificity). The area under the curve (AUC), calculated using logistic regression, was used to compare the diagnostic performance of the two tests; AUC values > 0.75 are generally considered to represent good performance. The pre-planned analysis was based on femoral neck BMD as in the original OSTA report, but results for lumbar spine BMD or total hip BMD are also presented. Sensitivity was defined as the proportion of women with osteoporosis (T-scores ≤ -2.5) that tested positive (OSTA ≤ -1), and specificity was defined as the proportion of women without osteoporosis who tested normal (OSTA > -1). The ROC curve was constructed, and the AUC and its 95% confidence interval (95% CI) were estimated by using SPSS statistical software 13.0 (SPSS, Inc.) and MedCalc v11.5.0.0 software. The prevalence of osteoporosis defined by DXA measurements according to the WHO criteria and the prevalence of painful new vertebral fractures were examined across different categories of the OSTA risk index. A *p* value less than 0.05 was considered statistically significant.

## Results

A sample of 1320 postmenopausal women in Beijing participated in this study. In accordance with the exclusion criteria, 119 subjects were excluded from the study, and a total of 1201 subjects were analyzed. The assessed subjects included 173 women who had suffered a painful vertebral fracture within 6 months before the BMD measurement and 1028 healthy subjects attending for routine screening without specific osteoporosis-associated symptoms.

The characteristics of the study population are summarized in Table 2. The average age of the women in the study was 62 years old (range: 45–89 years). Overall, the proportions of subjects found to have osteoporosis based on femoral neck BMD T-scores ≤ -2.5, total hip BMD T-scores ≤ -2.5, lumbar spine (L1–4) BMD T-scores ≤ -2.5 were 17.99%, 8.99%, and 20.57%, respectively. The mean body weight and mean body mass index (BMI) were 60 kg and 24 kg/m^2^, respectively. Based on the WHO criteria, the prevalence of osteoporosis (femoral neck, total hip, or spine BMD T-score ≤ -2.5) was 3.85% for ages 45–49 years (2 of 52), 13.86% for ages 50–59 (70 of 505), 30.91% for ages 60–69 (102 of 330), 55.17% for ages 70–79 (144 of 261), and 64.15% for ages ≥80 (34 of 53).

**Table 2:** **Summary of descriptive characteristics of the study cohort** (**n** = **1201**)

**Characteristic**	**Mean or value**	**Range**
Age (years, mean ± SD)	62.42 ± 9.27	45-89
Age group—n (%)		
<50 years	52 (4.33%)	
50–59 years	505 (42.05%)	50-59
60–69 years	330 (27.48%)	60-69
70–79 years	261(21.73%)	70-79
≥80 years	53 (4.41%)	80-89
Height (cm, mean ± SD)	158.64 ± 5.15	140-178
Weight (kg, mean ± SD)	60.47 ± 9.49	35-106
Body mass index (kg/m^2^, mean ± SD)	24.01 ± 3.50	14.34-39.41
BMD (g/cm^2^) (mean ± SD)		
L1–L4	0.83 ± 0.15	0.370-1.534
Femoral neck	0.67 ± 0.13	0.269-1.160
Total hip	0.77 ± 0.14	0.268-1.235
T-score (mean ± SD)		
L1–L4 T-score	-1.29 ± 1.44	-5.2–5.3
Femoral neck T-score	-1.34 ± 1.34	-5.5–3.6
Total hip T-score	-0.83 ± 1.27	-5.5–3.4
WHO diagnostic categories—n (%)^a^		
Normal	303/1201 (25.23)	
Osteopenia	546/1201 (45.46)	
Osteoporosis	352/1201 (29.31)	
T-score ≤-2.5—n (%)		
L1–L4	247/1201(20.57)	
Femoral neck	216/1201 (17.99)	
Total hip	108/1201 (8.99)	
History of fragility fracture—n (%)	341/1201(28.39)	
OSTA score (mean ± SD)	-0.35 ± 2.52	-10–8

^**a**^Lowest *BMD T*-score in the lumbar spine, femoral neck, or total hip was considered.

**Figure 1:**
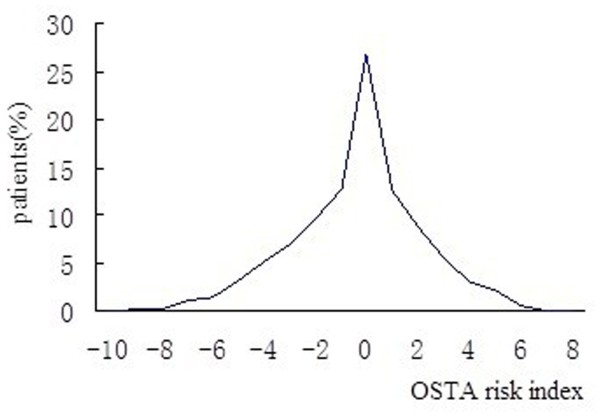
Distribution of study patients according to their OSTA risk index.

We then excluded the 173 women who had suffered a painful vertebral fracture within the 6 months preceding BMD measurement. Among the remaining 1028 subjects who attended for routine screenings without specific osteoporosis-associated symptoms, 229 (22.28%) had osteoporosis. The prevalence of osteoporosis was 3.85% (2/52) for patients in the 45–49 year-old age band, 13.05% (65/498) for patients in the 50–59 year-old age band, 26.39% (76/288) for patients in the 60–69 year-old age band, 44.97% (76/169) for patients in the 70–79 year-old age band, and 47.62% (10/21) for patients in the ≥ 80 year-old age band. Thus, the prevalence of osteoporosis increased progressively with age (χ^2^_trend_ = 9.63, *P* <0.001). The OSTA index varied from -10 to 8, and the percent distribution of the women according to the OSTA index is shown in Figure 1. Based on the osteoporosis risk categories used in Asian women [10], 59.45% of the women (n = 714, OSTA > -1) had a low risk, and 40.55% (n = 487, OSTA ≤ -1) of the women had an increased risk of osteoporosis. Approximately 28.39% (n = 341) of the studied women reported a history of low-trauma fracture (≥ 45 years, including those of the spine, hip, distal forearm, proximal humerus, and malleolus). Furthermore, among the 341 women with previous fragility fractures, 173 had suffered a painful vertebral fracture within 6 months before the BMD measurement, and 164 of these 173 women were confirmed to have recent painful osteoporotic vertebral fractures according to the clinical criteria mentioned above. Most women with painful vertebral fractures were admitted to Beijing Friendship Hospital and consented to PKP and osteoporosis treatments, including subcutaneous or intranasal rectal calcitonin and calcium and vitamin D supplementation. The remaining women who did not receive PKP were given osteoporosis treatments and traditional management including analgesia, rest with a corset support, and subsequent gradual mobilization within the limits of pain. Significant differences in age, BMI, BMD, T-scores, and OSTA variables were observed between the group of postmenopausal women with vertebral fractures and the group with no vertebral fractures (*P* < 0.01).

### Correlation between OSTA index values and BMD T-scores in postmenopausal women

**Figure 2:**
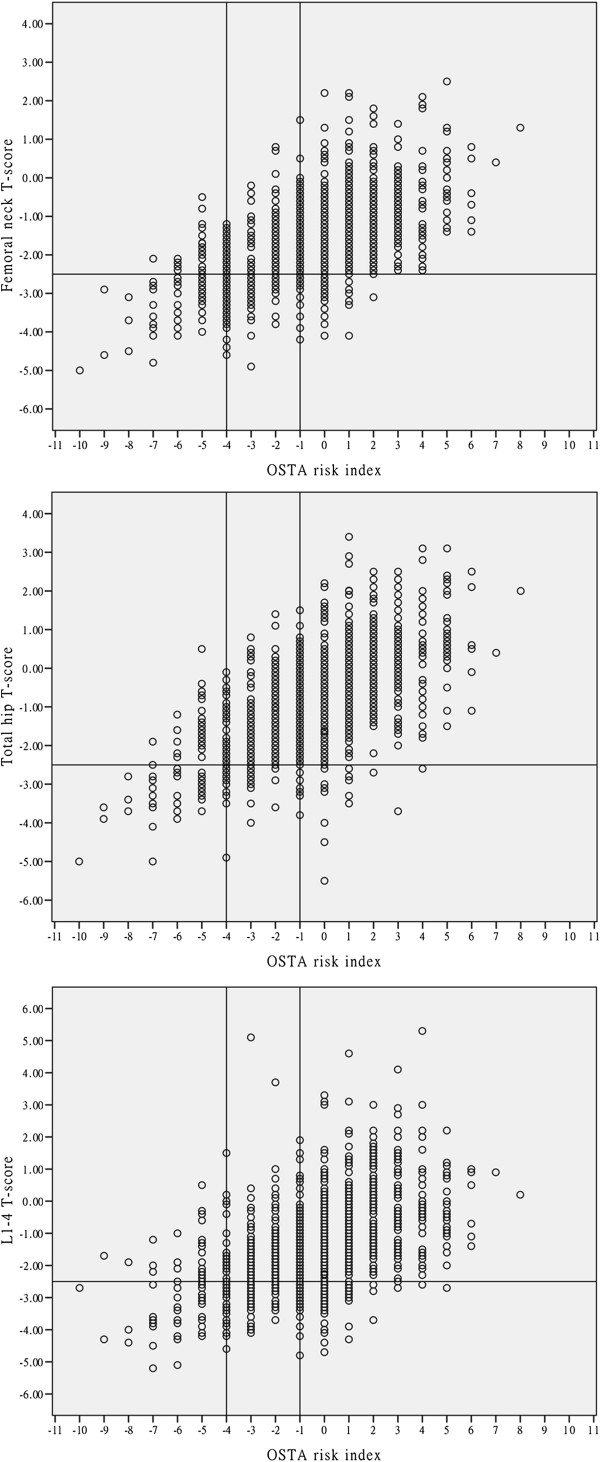
**Distribution of T**-**scores by OSTA index value.** The horizontal line demarcates a T-score of -2.5 and the vertical lines mark the OSTA risk index cutoffs of -1 and -4.

There was a moderate positive correlation between OSTA index values and BMD T-scores at different sites (femoral neck: r = 0.580, *P* < 0.001; total hip: r = 0.589, *P* < 0.001; L1–4: r = 0.489, *P* < 0.001). Figure 2 shows the distribution of T-scores at the femoral neck, total hip, and L1–L4 lumbar spine by the OSTA index values.

**Figure 3:**
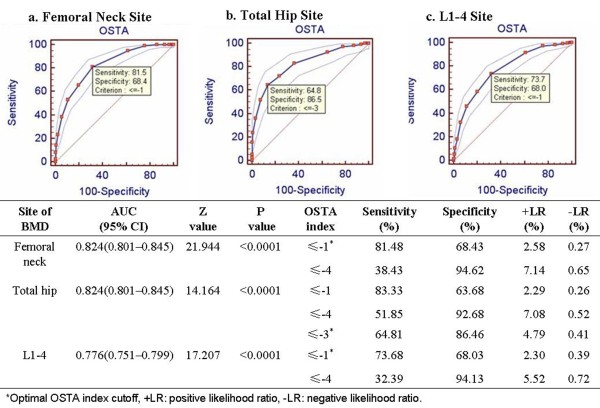
**AUC and sensitivity and specificity values of the OSTA index for the diagnosis of osteoporosis (T-score ≤ -2.5 SD) using BMD measurements at the femoral neck (a), total hip (b), and L1–L4 vertebrae (c).**

The area under the ROC curve for the OSTA index for the femoral neck, total hip, and L1–4 lumbar spine were 0.824 (*P* < 0.001), 0.824 (*P* < 0.001), and 0.776 (*P* < 0.001), respectively (Figure 3). Furthermore, the index cutoff of -1 provided a sensitivity of 74–83% and a specificity of 63–68%, whereas the sensitivity and specificity for an index cutoff of -4 were 32–52% and 93–95%, respectively (Figure 3). The optimal OSTA index cutoff at the femoral neck, total hip, and L1–4 lumbar spine were -1, -3, and -1, respectively (Figure 3).

As reported in detail in Table 3, we found that among the 1028 subjects who attended for routine screenings, the presence of osteoporosis was found in 44% and 11% of subjects in the increased risk group and low-risk group, respectively, when using BMD measurements at either the femoral neck or lumbar spine to diagnose osteoporosis. The sensitivity, specificity, positive predictive value (PPV) and negative predictive value (NPV) of the OSTA index for detecting osteoporosis were calculated for the overall population and by age groups. Among these women who attended for routine screenings, the sensitivity was 65.9% (151/229; 95% CI, 59.4-72.1), the specificity 75.5% (603/799; 95% CI, 72.3-78.4), PPV 43.5% (151/347; 95% CI, 38.2-48.9), and the NPV 88.5 (603/681; 95% CI, 85.9-90.8). The data analyzed by age groups showed that sensitivity and PPV increased with age, while specificity and NPV decreased with age.

**Table 3:** **Distribution of clinical index based on femoral neck** (**FN**), **total hip** (**TH**), **or lumbar spine BMDs in routine screening group**

**Group**	**OSTA value**	**FN, TH, or L1–4 BMD**		**Total**	**Sensitivity**		**Specificity**		**PPV**		**NPV**	
		**T ≤ -2.5**	**T > -2.5**		**%**	**95% CI**	**%**	**95% CI**	**%**	**95% CI**	**%**	**95% CI**
Routinely screened, all ages	High risk (≤ -1)	151	196	347	65.9	59.4–72.1	75.5	72.3–78.4	43.5	38.2–48.9	88.5	85.9–90.8
	Low risk (> -1)	78	603	681								
	Total	229	799	1028								
< 60 years	High risk (≤ -1)	20	43	63	29.9	19.3–42.3	91.1	88.2–93.5	31.7	20.6–44.7	90.3	87.4–92.8
	Low risk (> -1)	47	44	487								
	Total	67	483	550								
60–69 years	High risk (≤ -1)	52	75	127	68.4	56.7–78.6	64.6	57.8–71.0	69.3	57.6–79.5	85.1	78.6–90.2
	Low risk (> -1)	24	137	161								
	Total	76	212	288								
≥ 70 years	High risk (≤ -1)	79	78	157	91.9	83.9–96.7	25.0	17.0–34.4	50.3	42.2–58.4	78.8	61.1–91.0
	Low risk (> -1)	7	26	33								
	Total	86	104	190								

*PPV* positive predictive value, *NPV* negative predictive value.

### OSTA and the prevalence of vertebral fracture in postmenopausal women

**Figure 4:**
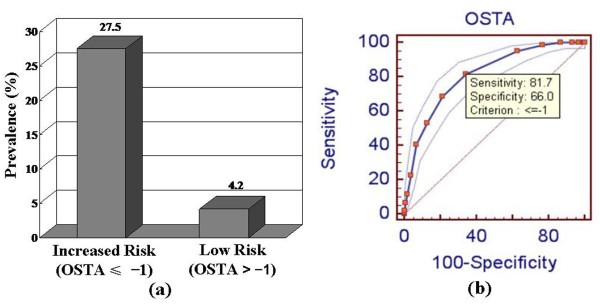
**Relation of OSTA to the prevalence of new vertebral fracture in the increased risk group and low risk group according to the OSTA classification scheme. (a)** Prevalence of new vertebral fracture by groups. **(b)** ROC curve.

With regard to new painful osteoporotic vertebral fractures, the area under the ROC curve (Figure 4) relating the OSTA index to identify vertebral fracture was 0.812 (95% CI: 0.789 to 0.834, Z = 18.592, *P* < 0.001). The optimal OSTA cutoff was -1.

## Discussion

Simple noninvasive diagnostic tools, such the OSTA, for detecting osteoporosis and predicting fracture risks in postmenopausal women are very important, particularly in places, such as China, where DXA technology is not wide-spread. In this study, the use of the OSTA tool was found to have high sensitivity and good specificity for identifying primary osteoporosis in postmenopausal Han Chinese women in Beijing. More importantly, we found that this tool may be useful for identifying new clinical vertebral fractures.

Compared to the majority of previous studies, this study had several noteworthy strengths. Firstly, our study was not retrospective. Both healthy women who came to the hospital for health examinations and clinical patients were selected for this study according to strict clinical criteria. All of the subjects’ weights and heights were measured at the same time as that BMD measurements were taken. Secondly, we built strict inclusion and exclusion criteria to exclude the effects of secondary osteoporosis, nationality, and any antiresorptives/anabolic agents. All women were Beijing citizens with long-term residency in Beijing. Thirdly, most previous studies defined osteoporosis by a T-score ≤ -2.5 only at the femoral neck. In this study, we analyzed the value of OSTA for identifying osteoporosis based on the WHO criteria (T-score ≤ -2.5 at the femoral neck, total hip, or lumbar spine), which may be more practical because it is widely accepted that osteoporosis patients diagnosed according to the WHO criteria should receive specific bone active treatments, such as antiresorptive or anabolic agents. Furthermore, for the first time, we evaluated the value of OSTA for identifying recently acquired symptomatic clinical vertebral fractures, which were confirmed by reasonable clinical criteria. As one of the largest general hospitals in Beijing, our patient population had a wide representation in terms of age, height, weight, and BMD status.

This study was the first to analyze the correlation between the OSTA index and BMD T-scores at different sites. The results indicated a significant positive correlation. Furthermore, we started to validate the usefulness of the OSTA in the studied cohort. An index cutoff of -1 provided high sensitivity (74–83%) and good specificity (63–68%) for identifying subjects with a BMD T-score ≤ -2.5 at different skeletal sites relative to previously reported data [9-13]. The optimal OSTA cutoff may vary with different skeletal sites. The AUC for OSTA was approximately 0.8 at the different skeletal sites. The results were similar to a previous study in a southern Chinese cohort [11] in which 259 of 487 women with an OSTA index ≤ -1 (increased risk group) and only 93 of 714 women with OSTA index > -1 (low risk group) were diagnosed with osteoporosis in accordance with the WHO criteria. Among 1028 women aged 45 years or older who came for routine screening, using a cutoff of OSTA≤ -1, we obtained sensitivity of 65.9%, specificity of 75.5%, a PPV of 43.5%, and an NPV of 88.5. Although the PPV was only 43.5%, more importance should be given to specificity than sensitivity. A high specificity and a low positivity rate of OSTA index can be translated into a low referral rate for BMD measurement by DXA. With these rates, only high-risk women (OSTA≤ -1) would be referred to physicians because of OSTA’s high specificity, especially among postmenopausal women younger than 60 years of age, yielding a specificity greater than 90%. Referring only high-risk women could potentially enhance resource use efficiency. BMD measurements in low-risk women can be avoided and proper medical care in high-risk women encouraged. Moreover, there is no risk of harm to the patient from unnecessary treatment or invasive diagnostic testing in the case of a false-positive result from the OSTA.

Several studies show that OSTA is an effective method for identifying people at low risk of osteoporosis [12,13]. Our data showed that the OSTA could identify women at low risk of osteoporosis for whom DXA testing was unnecessary. The NPV was 88.5% in the overall population and 90.3% in postmenopausal women under 60 years of age. PPV of OSTA increased with age, while NPV decreased with age. However, NPV was only 78.8% among women ≥ 70 years. Therefore, we can recommend that high risk women younger 70 years of age be referred for BMD measurement, while all women 70 years old or older be referred for BMD measurement due to the high prevalence of osteoporosis and poor NPV among women in this age group.

It is widely accepted that postmenopausal women with fragility fractures have a high risk of subsequent fractures, and are therefore eligible for treatment to reduce the risk of future fractures [16-18]. Previous studies showed that the OSTA may be a simple and effective tool for identifying postmenopausal women at increased risk of nonvertebral fractures and vertebral deformity [5,19]. However, it was not clear whether the OSTA index could identify painful new vertebral fractures. In our study population, we assessed the ability of OSTA to identify postmenopausal women with painful new vertebral fractures. All 164 painful new vertebral fractures were confirmed by reasonable clinical criteria, and most of the patients accepted PKP treatment. Sensitivity and specificity values of 82% and 67%, respectively, were obtained with an OSTA cut-off value ≤ -1. The area under the ROC curve relating the OSTA index to new vertebral fractures was 0.70. The prevalence of vertebral deformities according to the OSTA index was 27.5% (134/487) in the increased risk group, and 4.2% (30/714) in the low risk group. Our findings showed the OSTA index may be a useful tool to screen for new painful vertebral fractures.

The present study has several limitations. First, the subjects who were recruited from an osteoporosis center in a general hospital could not fully represent the actual female population in Beijing. According to population census in 2011, the women in the 50–59-year-old, 60–69-year-old, 70–79-year-old, and ≥ 80 year-old age bands accounted for 61%, 23%, 13%, and 4% of the total women aged 50 years or older, respectively. The population structure of our study was different from the actual demographic situation in Beijing, which could impact the generalizability of these data. However, the clinical performance of OSTA was analyzed by age group, and our conclusions were based on this analysis. Secondly, we could not assess the value of OSTA for identifying painful nonvertebral fractures in this study. Thirdly, our results should be confirmed in other cohorts.

## Conclusion

In summary, the present study showed that in postmenopausal Han Chinese women in Beijing, the OSTA index may be a simple and effective clinical risk assessment tool for identifying the risk of osteoporosis as defined by DXA according to the WHO diagnostic criteria, and it may be a useful tool for identifying new painful vertebral fractures. Given the simplicity and validity of the OSTA index and the benefits shown in our study, we can recommend it as an important health-promotion activity to women, especially postmenopausal women under 70 years of age.

## Competing interests

The authors declare that they have no competing interests.

## Authors’ contributions

QF is the principal investigator, project design and direction, preparation and review of the manuscript. YY and BQW coordination field work, preparation and review of the manuscript. QM coordination and analysis of DXA BMD measurements, review of the manuscript. DL coordination and management of the cohort, review of the manuscript. HT and JJL scientific support and methodological expert, review of the manuscript. NS statistical analysis and management of the database, review of the manuscript. All authors read and approved the final manuscript.

## Pre-publication history

The pre-publication history for this paper can be accessed here:

http://www.biomedcentral.com/1471-2474/14/271/prepub

## References

[B1] Cummings SR Melton LJ Epidemiology and outcomes of osteoporotic fractures Lancet 2002 359 1761 1767 10.1016/S0140-6736(02)08657-9 12049882

[B2] Cooper C Campion G Melton LJ 3rd Hip fracture in the elderly: a worldwide projection Osteoporos Int 1992 2 285 289 10.1007/BF01623184 1421796

[B3] Hochberg M Preventing fractures in postmenopausal women with osteoporosis: a review of recent controlled trials of antiresorptive agents Drugs Aging 2000 17 317 330 10.2165/00002512-200017040-00007 11087009

[B4] Siris E Alendronate in the treatment of osteoporosis: a review of the clinical trials J Women’s Health Gend Med 2000 9 599 606 10.1089/15246090050118125 10957748

[B5] Tao B Liu JM Li XY Wang JG Wang WQ Ning G An assessment of the use of quantitative ultrasound and the osteoporosis self-assessment tool for Asians in determining the risk of nonvertebral fracture in postmenopausal Chinese women J Bone Miner Metab 2008 26 60 65 10.1007/s00774-007-0798-0 18095065

[B6] Burger H van Daele PLA Algra D van den Ouweland FA Grobbee DE Hofman A van Kuijk C Schutte HE Birkenhager JC Pols HA The association between age and bone mineral density in men and women aged 55 years and over: the Rotterdam study J Bone Miner Res 1994 25 1 13 10.1016/S0169-6009(08)80203-6 8061547

[B7] Edelstein SL Barett-Connor E Relation between body size and bone mineral density in elderly men and women Am J Epidemiol 1993 138 160 169 8356959 10.1093/oxfordjournals.aje.a116842

[B8] Hannan MT Felson DT Anderson JJ Bone mineral density in elderly men and women: results from the Framingham osteoporosis study J Bone Miner Res 1992 7 547 553 1615761 10.1002/jbmr.5650070511

[B9] Nguyen TV Center JR Pocock NA Eisman JA Limited utility of clinical indices for the prediction of symptomatic fracture risk in postmenopausal women Osteoporos Int 2004 15 49 55 10.1007/s00198-003-1511-3 14593453

[B10] Koh LK Sedrine WB Torralba TP Kung A Fujiwara S Chan SP Huang QR Rajatanavin R Tsai KS Park HM Reginster JY A simple tool to identify Asian women at increased risk of osteoporosis Osteoporos Int 2001 12 699 705 10.1007/s001980170070 11580084

[B11] Kung AW Ho AY Sedrine WB Reginster JY Ross PD Comparison of a simple clinical risk index and quantitative bone.ultrasound for identifying women at increased risk of osteoporosis Osteoporos Int 2003 14 716 721 10.1007/s00198-003-1428-x 12897978

[B12] Park HM Sedrine WB Reginster JY Ross PD Korean experience with the OSTA risk index for osteoporosis: a validation study J Clin Densitom 2003 6 247 250 10.1385/JCD:6:3:247 14514994

[B13] Yang NP Lin T Wang CS Chou P Correlation of osteoporosis screening by quantitative ultrasound of calcaneus and osteoporosis self-assessment tool for Asians in Taiwanese J Formos Med Assoc 2004 103 130 136 15083244

[B14] Lu CY Chen DC Cai YH Wei SQ Concordane of OSTA and lumbar spine BMD by DXA in identifying risk of osteoporosis J Orthop Surg 2006 1 14 10.1186/1749-799X-1-14 PMC1693545 17150121

[B15] Kanis JA on behalf of the World Health Organization Scientific Group Assessment of osteoporosis at the primary healthcare level. Technical Report 2008 UK: WHO Collaborating Centre, University of Sheffield

[B16] Klotzbuecher C Ross P Landman P Abbott TA III Berger M Patients with prior fractures have an increased risk of future fractures: a summary of the literature and statistical synthesis J Bone Miner Res 2000 15 721 739 10780864 10.1359/jbmr.2000.15.4.721

[B17] Meunier PJ Delmas PD Eastell R McClung MR Papapoulos S Rizzoli R Seeman E Wasnich RD Diagnosis and management of osteoporosis in postmenopausal women: guidelines for clinicians Clin Ther 1999 21 1025 1044 10.1016/S0149-2918(99)80022-8 10440625

[B18] Freedman KB Kaplan FS Bilker WB Strom BL Lowe RA Treatment of osteoporosis: are physicians missing an opportunity? J Bone Joint Surg Am 2000 82 1063 1070 10954094 10.2106/00004623-200008000-00001

[B19] Saetung S Ongphiphadhanakul B Rajatanavin R The relationship of an Asian-specific screening tool for osteoporosis to vertebral deformity and osteoporosis J Bone Miner Metab 2008 26 47 52 10.1007/s00774-007-0796-2 18095063

